# Effectiveness of Early Warning Scores for Early Severity Assessment in Outpatient Emergency Care: A Systematic Review

**DOI:** 10.3389/fpubh.2022.894906

**Published:** 2022-07-14

**Authors:** Amaya Burgos-Esteban, Vicente Gea-Caballero, Patricia Marín-Maicas, Azucena Santillán-García, María de Valvanera Cordón-Hurtado, Elena Marqués-Sule, Marta Giménez-Luzuriaga, Raúl Juárez-Vela, Juan Luis Sanchez-Gonzalez, Jorge García-Criado, Iván Santolalla-Arnedo

**Affiliations:** ^1^Government of La Rioja, Rioja Health Service Servicio Riojano de Salud, La Rioja, Spain; ^2^Department of Nursing, Research Group in Care Grupo de Investigación en Cuidados, University of La Rioja, Logroño, Spain; ^3^Patient Blood Management Research Group, Madrid, Spain; ^4^Community Health and Care Research Group, Faculty of Health Sciences, Valencian International University, Valencia, Spain; ^5^Castilla-Leon Health Service, Sanidad Castilla y Leon, University Hospital of Burgos, Burgos, Spain; ^6^Department of Physiotherapy, University of Valencia, Valencia, Spain; ^7^Department in Nursing and Physiotherapy, University of Salamanca, Salamanca, Spain; ^8^Department of Physiology and Pharmacology, Faculty of Medicine, University of Salamanca, Salamanca, Spain; ^9^Castilla-Leon Health Service, Sanidad Castilla y Leon, University Hospital of Salamanca, Salamanca, Spain

**Keywords:** emergency medicine, Emergency Medical Service (EMS), medicine, emergency care, scale

## Abstract

**Background and Objectives:**

Patient assessment and possible deterioration prediction are a healthcare priority. Increasing demand for outpatient emergency care services requires the implementation of simple, quick, and effective systems of patient evaluation and stratification. The purpose of this review is to identify the most effective Early Warning Score (EWS) for the early detection of the risk of complications when screening emergency outpatients for a potentially serious condition.

**Materials and Methods:**

Systematic review of the bibliography made in 2022. Scientific articles in Spanish and English were collected from the databases and search engines of Pubmed, Cochrane, and Dialnet, which were published between 2017 and 2021 about EWSs and their capacity to predict complications.

**Results:**

For analysis eleven articles were selected. Eight dealt with the application of different early warning scores in outpatient situations, concluding that all the scoring systems they studied were applicable. Three evaluated the predictive ability of various scoring systems and found no significant differences in their results. The eight articles evaluated the suitability of NEWS/NEWS2 to outpatient conditions and concluded it was the most suitable in pre-hospital emergency settings.

**Conclusions:**

The early warning scores that were studied can be applied at the pre-hospital level, as they can predict patient mortality in the short term (24 or 48 h) and support clinical patient evaluation and medical decision making. Among them, NEWS2 is the most suitable for screening potentially deteriorating medical emergency outpatients.

## Introduction

Scientific evidence shows that patient deterioration can be predicted from 6 to 24 h in advance (1–3). Sudden changes in heart rate, arterial systolic blood pressure, respiratory rate, temperature, oxygen saturation, or level of consciousness, happen moments before the clinical deterioration of the patient (1). Given this evidence, the European Resuscitation Council published Guidelines in 2021 requiring hospitals to have an early warning scoring system in place to identify patients whose state of health may suffer imminent deterioration. Correct evaluation of patients through these systems should be complemented with specially trained personnel qualified to provide a prompt response, reducing mortality from cardiac arrest in hospitalized patients (4, 5). A significant number of preventable mortality cases could be avoided by implementing early warning scores and quick response systems, as previous patient deterioration is not detected in up to 31% of preventable inpatient mortality (6).

The target is identical when it comes to outpatients; however, there are very limited diagnostic tools available in this setting. A rating scale easy to apply would help clinics make better decisions when determining patients with a higher deterioration chance, resulting in better care and the prevention of complications (7).

There are numerous early warning scores of the risk of complications, up to 100, used in different countries since the late 90's to evaluate patient conditions (8). They carry out early detection of clinical deterioration, thus facilitating the activation and intervention of response teams and enabling a quick transfer to intensive care units, which improves the prognosis and chances of survival (9). These tools measure a set of physiological parameters that are objectively standardized and validated (Annex 1) (3, 10).

Assessing the patient is an essential step in early deterioration detection both in and out of the hospital. A correct assessment will achieve two goals. First, providing the patient with a greater level of care, thus preventing deterioration, and promoting an earlier recovery. The second goal is a direct consequence of the first, i.e. greater system efficiency, since reducing morbidity will lead to shorter hospital stays and less health spending, while always guaranteeing the best quality of care (1, 4, 5).

Spain's growing demand for healthcare by using the 112/061 emergency numbers (11) requires the establishment of an effective and validated care prioritization system, which should fulfill two purposes. One is to facilitate the decision-making process of the doctors and nurses of the Coordination Centers for Urgencies and Emergencies assessing the conditions of patients calling from home and mobilizing the appropriate health care resources in the shortest time possible (7, 12–14). The other purpose is to facilitate the decision-making process in the triage and assignment of patients arriving at the hospital, providing a comprehensive and reliable assessment that will expedite the care process, reducing waiting time and promoting quick patient care (15–17). Non-invasive pre-hospital monitoring of the parameters needed to establish the use of validated rating is simple, and it could improve the chances of early patient deterioration detection (1, 7, 12).

The above will affect the quality of care and the satisfaction level of the population receiving it (10, 18) as far as perceived patient safety (10, 13, 16, 19). These two concepts are most important in current health management for improving healthcare effectiveness and efficiency.

Regarding the recommendation established by the European Resuscitation Council Guidelines (2021), it seems relevant to examine the literature on the properties of the scales currently used, both in and out-of-hospital. This article will make available to healthcare professionals a document that summarizes the most current evidence and will enable clinical decision making. It will be particularly relevant for optimizing the detection and management of potentially severe patients. In addition, it will be innovative for outpatients, as the available evidence in this setting is more limited.

Based on the above, the aim of this study is to identify the most effective early detection score of the risk of complications in potentially serious medical conditions of emergency outpatients.

## Materials and Methods

Method: systematic review of the literature created from September 2021 to January 2022 according to PRISMA statement guidelines (20).

Review and search: five of the authors participated in the search of literature available in Spanish and English from 2017 to 2021 relating to early warning scores applied to the assessment of adult patients (≥18 years), using Pubmed, Cochrane, and Dialnet search engines and data.

Inclusion criteria: cross-sectional descriptive scientific articles, case series, randomized clinical essays, and systematic reviews including bibliography generally showing the use of validated scores; articles referring to the predictive ability of various scores.

Exclusion criteria: articles collecting editorials, clinical notes, and letters to the editor; articles referring to care for pregnant women; articles about scores designed to assess the severity of specific conditions (sepsis, trauma, covid); articles on early warning scores with a single parameter.

Search strategy: a researcher did the initial search; two authors carried out the selection of articles independently; subsequently, the studies selected by each of the reviewers were reassessed for inclusion, with a third reviewer resolving discrepancies. Two authors selected the variables and evaluated the quality of the articles selected, while a third researcher handled any discrepancies. The search was completed by “reverse search”; the 2021 Executive Summary and Guidelines of the European Resuscitation Council were consulted, together with the 2020 Cardiopulmonary Resuscitation Guidelines of the American Heart Association, in addition to the Spanish legislation in force and Ministry of Health statistics portal, to contextualize the current situation in Spain. The following natural language terms were searched: Early warning scores, Pre-hospital setting, Deteriorating patients. The following MeSH terms were searched: Early warning score, Emergency Medical Services. Logical relations were established between these terms using the Boolean operators AND to narrow the search, and OR to broaden it. The search strategy (Table 1) was based on the following research question raised in the review and made using the format PICO (22): What is the most effective early warning score in outpatient settings to assess patients with potentially serious conditions and early deterioration detection? (Table 2).

**Table 1:** Search strategy.

**Database**	**Search strategy**	**Results**	**Selected**	**References**
Pubmed	((“Emergency Medical Services”[Mesh]) AND (early warning score [MeSH Terms])	58	7	(13–16, 19, 21)
	(“Early Warning Score”[Mesh] AND (prehospital setting)	13	5	(9, 12–14, 19)
	“Early Warning Score”[Mesh] AND (meta-analysis [Filter] OR randomizedcontrolledtrial[Filter] OR systematicreview[Filter]) AND (systematicreview[Filter])	16	1	(17)
	Early Warning Score AND ((y_5[Filter]) AND (meta-analysis [Filter] OR systematicreview[Filter]) AND (alladult[Filter]))	6	0	
	Early warning score AND deteriorating patients AND pre-hospital setting	5	1	(15)
Dialnet	Early warning scores	19	2	(10, 12)
Cochrane	“Early warning score” AND “prehospital setting”	17	0	

**Table 2:** Research question in PICO format.

**Patient**	**Intervention**	**Comparison**	**Result**
Potentially serious patients	Assessment using most effective early warning score in outpatient setting	Effectiveness of different early warning scores	Early deterioration detection

Quality assessment: CASPe (23) critical appraisal and STROBE (24) statement checklists were used, according to the type of study evaluated. Compliance with 70% of the items evaluated was established as the minimum quality criterion to include an article in the study.

Data collection: a previously designed template was used to collect the following data: author, year, type of study, methodological quality (checklist and result obtained), population/sample, early warning score(s) evaluated in the study, score effectiveness, and outpatient validation. Score effectiveness was defined as the capacity to predict patient mortality within 24 or 48 h.

Research variables: short-term prediction capacity (24 or 48 h); pre-hospital application of early warning scores; early warning scores validated for the outpatient setting.

**Figure 1:**
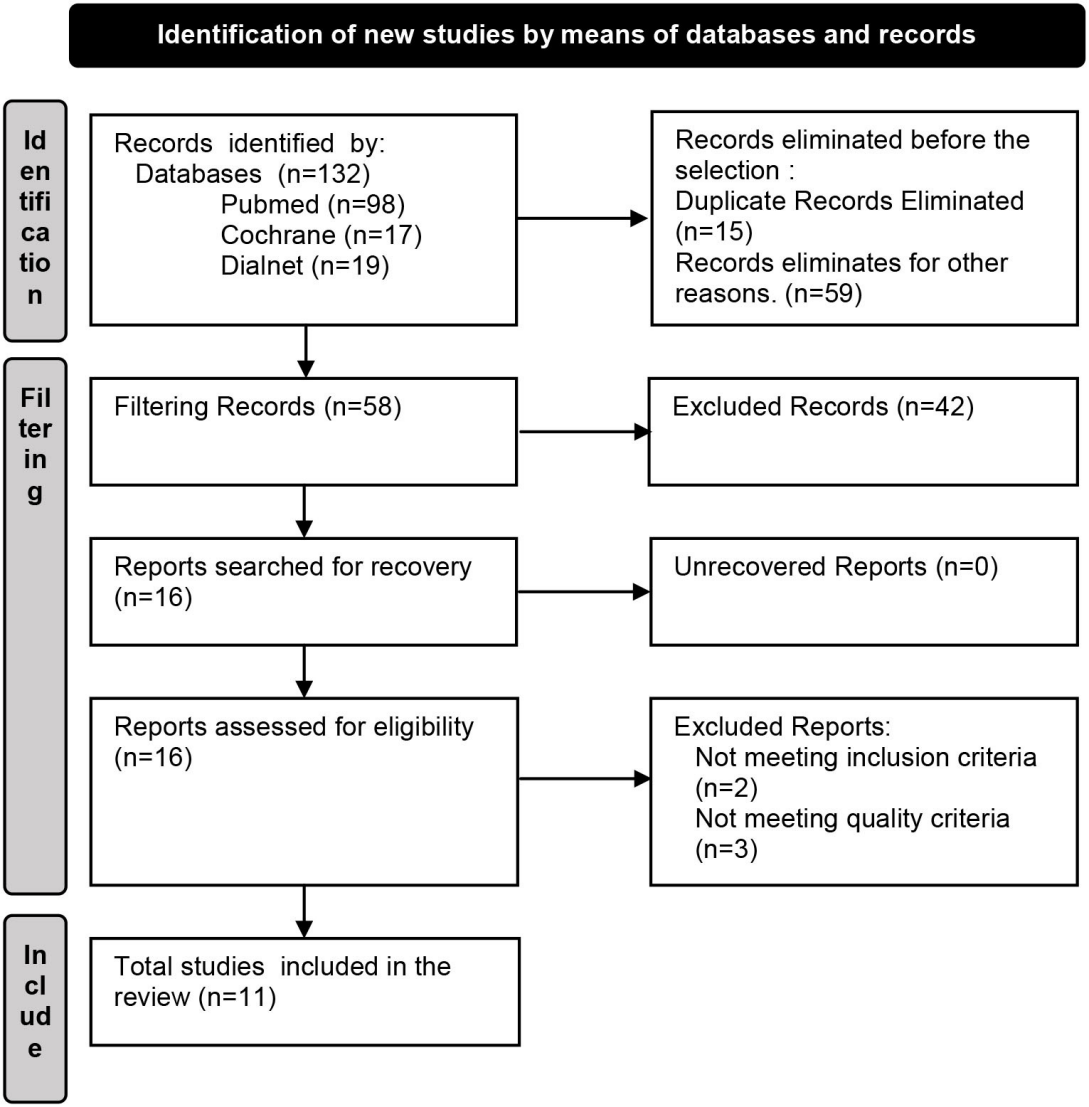
Flowchart.

Identification of articles: 132 articles were identified initially (Pubmed 98, Dialnet 19 and Cochrane 17). Upon the removal of duplicates (15 articles) and those not conforming with the established criteria (59 articles), we proceeded with reading the title summary of the remaining (58 articles). After checking inclusion and exclusion criteria, 16 studies were finally selected for eligibility assessment. After a critical review, 5 articles were eliminated, 3 of them because they did not meet the quality criteria, and the other 2 because they did not satisfy the inclusion criteria in the end. After a detailed process of localization, choice, and inclusion, 11 articles were selected for inclusion in the study. This process is summarized in the annexed flowchart (Figure 1).

## Results

Eleven studies were included in the review, published between 2017 and 2021. Three of these were observational studies (9, 12, 13), three were systematic reviews (10, 15, 17), one featured a meta-analysis (15), and the remaining five were cohort studies (7, 14, 16, 19, 21), two of them prospective (7, 16) and three retrospective (14, 19, 21). As for the country of publication, five were published in Spain (7, 9, 10, 12, 13), two in the United Kingdom (15, 17), two in Finland (19, 21), one in Australia (16), and one in Japan (14). Eight of the articles focused on the application of early warning scores at the outpatient level (7, 9, 12–15, 19, 21). The most cited were the National Early Warning Score (NEWS) and its 2017 update NEWS2, referred to in all eleven studies. Five articles cited the VitalPAC Early Warning Score (ViEWS) (9, 10, 13, 16) or Prehospital VitalPAC Early Warning Score (PhViEWS) (15), and the Modified Early Warning Score (MEWS) (9, 10, 13, 15, 16); other scores were only cited in three or fewer articles. The main goal of eight of the studies was to assess the short-term prediction ability of various scores as far as mortality within 24 or 48 h (9, 10, 12, 13, 16, 17, 19, 21). The goal of two of the articles was to determine deterioration prediction capacity (7, 15). Three had the main purpose of determining both short-term mortality and deterioration prediction capacity (10, 16, 17). Three sought to determine the applicability of early warning scores in a pre-hospital setting (9, 13, 14).

### Short-Term Prediction Ability (24 or 48 h)

After analyzing prediction capacity, we concluded that most of the EWS scores were good or excellent predictors of short-term mortality (9, 16). The ViEWS score stood out as the most predictive, followed by NEWS and AbViEWS (9). One of the studies (17) specified that NEWS was better to identify patients at a greater risk of mortality within 24 h. The authors emphasized the importance of complying with the EWS application protocols and activating Quick Response Teams to obtain the highest effectiveness from these systems. Two of the articles analyzed (7, 19) agreed in affirming that NEWS (NEWS2) showed a high short-term mortality prediction ability. Two studies considered the possibility of increasing NEWS prediction capacity by adding the capillary glucose figure or that of lactate serum. One of them compared the prediction capacity of NEWS and NEWS-gluc (capillary glucose determination added), establishing that the NEWS-gluc calculation had a slightly higher ability to identify risk than NEWS (21). The other assessed whether the addition of lactate serum to pre-hospital NEWS might improve early mortality prediction, reaching the conclusion that there were no significant differences between NEWS and NEWS-L in this regard (12).

### Pre-hospital Application of Early Warning Scores

The scores NEWS2, MEWS, ViEWS, TREWS, WPSS (Worthing Physiological Scoring System, MREMS (Modified Rapid Emergency Medicine Score) (25), and PI (Prehospital Index) (26) are clinical tools that can help decision making at a critical time and whose use would help standardize early deterioration detection in the outpatient setting (13). The EWS, MEWS, HEWS, ViEWS, SEWS, and NEWS2 systems are suitable for pre-hospital use due to their ease of application in this setting (9); however, patient assessment using these scores should never replace objective clinic evaluation, as the two should be complementary (9, 15). In the United Kingdom, the NEWS score is widely used in the pre-hospital setting (15).

After testing the validity of NEWS2, MEWS, ViEWS, WPSS (Worthing Physiological Scoring System), TREWS, MREMS (Modified Rapid Emergency Medicine Score), and PI (Prehospital Index) in the pre-hospital setting, it was established that there were no significant differences between them (13). The use of NEWS2 was justified because it is a tool validated for pre-hospital use that offers advantages from the clinical point of view (it evaluates the supply of oxygen to the patient), and multiple studies confirm its usefulness. Applied to the outpatient setting, NEWS2 was established to be an excellent predictor of which patients have a greater possibility of mortality in the short term, while also emphasizing the importance of facilitating patient assessment considering the limited resources available in this setting (7, 14, 19). The calculation of NEWS-gluc (21) and NEWS-L (12) at the outpatient level is possible if both parameters are easily determined and managed even in outpatient locations.

### Early Warning Scores Validated for the Outpatient Setting

Our 11-article review observed the use of 25 early warning scores: EWS, NEWS2 (NEWS), NEWS-gluc, NEWS2-L, MEWS, PMEWS, MEWS GCS, ViEWS, PhViEWS, AbViEWS, WPPS, TREWS, REMS, MREMS, PI, HEWS, SWES, PRS, NzNEWS, RAPS, P. GOODACRE, P. GROARKE, GAP, VSS, and VSG. We were able to verify the validation at the pre-hospital level for seven of them: NEWS2 (NEWS), MEWS, ViEWS, WPPS, TREWS, MREMS, and PI.

The analysis of the 11 articles (Table 3) selected shows that the NEWS2 score (NEWS) is a useful, simple, and effective tool applicable in any setting, for both systematic patient assessment and pre-hospital healthcare (7, 12, 13, 27).

**Table 3:** Results.

**Author, Year**	**Population / sample**	**Plan**	**Score assessed**	**Score effectiveness** **(24 h)**^**1**^ **(48 h)**^**2**^	**Outpatient validation**	**Methodological quality**
Martín-Rodríguez et al. (7)	2,335	Multicentre prospective cohort study	NEWS 2	AUC 0.862^**1**^	SI	9/11^***^
Martín-Rodríguez et al. (9)	349	Prospective longitudinal observational study	EWS MEWS HEWS ViEWS SWES NEWS2	AUC 0.885^**2**^ AUC 0.848^**2**^ AUC 0.890^**2**^ AUC 0.894^**2**^ AUC 0.884^**2**^ AUC 0.896^**2**^	SI SI SI SI SI SI	17/22^*^
Arévalo-Buitrago et al. (10)	165,580	Systematic review and meta-analysis	NEWS2 MEWS REMS TREWS SEWS ViEWS	AUC 0.883^**1**^**;** 0.8867^**2**^ ^**−**^ ^**−**^ ^**−**^ ^**−**^ ^**−**^	SI SI - - SI SI	9/10^**^
Martín-Rodríguez et al. (12)	707	Observational, prospective, and longitudinal study	NEWS2-L NEWS 2	AUC 0.91^**2**^ AUC 0.90^**2**^	- SI	20/22^*^
Martín-Rodriguez et al. (13)	3,273	Prospective multicentre observational cohort study	NEWS 2 MEWS ViEWS WPPS TREWS MREMS PI	AUC 0.861^**1**^**;** 0.86^**2**^ AUC 0.848^**1**^; 0.846^**2**^ AUC 0.873^**1**^; 0.862^**2**^ AUC 0.861^**1**^; 0.864^**2**^ AUC 0.871^**1**^; 0.868^**2**^ AUC 0.867^**1**^; 0.864^**2**^ AUC 0.831^**1**^; 0.827^**2**^	SI SI SI SI SI SI SI	10/11^***^
Takuro Endo et al. (14)	2,847	Observational retrospective cohort study	NEWS	AUC 0.90^**1**^	SI	9/11^***^
Rita Patel et al. (15)	157,878	Systematic review	NEWS MEWS PMEWS PRS NzNEWS PhNEWS	- - - - - -	SI SI - - - -	9/10^**^
William Spencer et al. (16)	690	Prospective cohort study	RAPS MEWS MEWS GCS REMS P. GOODACRE WPS P. GROARKE ViEWS/AbViEWS GAP VSS NEWS VSG	AUC 0.81^**2**^ AUC 0.91^**2**^ AUC 0.91^**2**^ AUC 0.83^**2**^ AUC 0.78^**2**^ AUC 0.90^**2**^ AUC 0.89^**2**^ AUC 0.96^**2**^/0.95^**2**^ AUC 0.81^**2**^ AUC 0.86^**2**^ AUC 0.95^**2**^ AUC 0.67^**2**^	SI - - - - - SI - - SI -	10/11^***^
Nicola Credland et al. (17)		Systematic review	NEWS/NEWS2	AUC 0.894	SI	9/10^**^
Pirneskoski, et al. (19)	35,800	Retrospective cohort study	NEWS	AUC 0.840^**1**^	SI	10/11^***^
Vihonen et al. (21)	27,141	Retrospective cohort study	NEWS-gluc NEWS	AUC 0.851^**1**^ AUC 0.844^**1**^	- SI	9/11^***^

* *STROBE statement checklist of essential points that should be described when publishing observational studies.*

** *CASPe critical appraisal checklist to help understand a systematic review*.

*** *CASPe critical appraisal checklist to help understand a cohort study*.

## Discussion

The present study allowed us to summarize the existing information on healthcare use of early warning scores of the risk of complications. These scores were initially designed for hospital use. There is a great deal of scientific evidence on the suitability of EWS to detect patients with greater chances of short-term mortality (24 or 48 h) (16). This motivated an investigation on the convenience of applying these scores to other healthcare levels, including pre-hospital (9, 13, 16). The growing demand for healthcare through Emergency Medical Services (EMS)—the Ministry of Health statistics portal recorded 9,084,399 requests in 2020—led to the mobilization of 4,611,404 aid resources by land and air (6). As a result, it is essential to establish a rating system to help identify users who need immediate attention. It is paramount to identify the early warning score of the risk of complications considered the most effective for screening potentially serious conditions in emergency service patients.

Our study shows that many of the early warning scores of risk are reliable tools; most of them obtained results showing a great ability to predict short-term mortality, including in pre-hospital settings (14). They are quick and easy to apply, which is very important in outpatient settings, where the available time and adverse conditions of patient care are usually unfavorable and the need to make quick decisions with very limited information is a constant in the day-to-day work of the staff in these services. Therefore, we can agree that most of the EWS scores allow us to identify critical and potentially critical patients and assess the seriousness of their clinical situation, which facilitates the decision-making process and quick response of care teams (15). They are also easily applicable in outpatient settings, although not all of them are validated for this use (9, 13). The standardization of an EMS patient assessment system will be useful to administrative management, insofar as it will facilitate decisions for either hospital admission or home care, choosing the best device to transfer a patient (7, 19), or making a pre-alert call to the hospital (9), in addition to making clinical decisions regarding the most appropriate patient treatment (9, 15). Most importantly, it is a tool that will provide objectivity in decision-making, thus ensuring that the intervention on the patient is the same regardless of the professional providing the care. The application of the scale would lead all professionals to make the same decision regarding the need for transfer and the most appropriate treatment.

The Spanish emergency system, through the 112 and 061 services, is equipped with the personnel (physicians, nurses, emergency health technicians and teleoperators, announcers and administrative assistants) and mobile devices (A1, B and C ambulances, emergency air teams, rapid intervention vehicles and special disaster vehicles) (11) necessary to implement an assessment system using an early assessment scale.

This study was also intended to establish which of the early warning scores of risk is more effective for the detection of mortality within 24 or 48 h. Data comparison evidenced that all the scores have great prediction capacity for short-term death, as shown by the AUC figures being between 0.90 (CI 95% 0.87–0.93) for NEWS at 24 h recorded in the study of Takuro Endo et al. (14) and 0.831 (CI 95% 0.78–0.87) for Prehospital Index at 24 h recorded in the study by Martín-Rodríguez et al. (13). From this we can determine that the most effective score to predict the chance of mortality within 24 h is NEWS/NEWS2, and that Prehospital Index is the least effective.

As for the 48 h mortality prediction period, the situation is similar, NEWS2-L and NEWS2 show the highest ability in the study by Martín-Rodríguez et al. (12), followed by MEWS according to another study by Martín-Rodríguez et al. (9), PI being the lowest in this case too, Martín-Rodríguez et al. (13). The effectiveness of early warning scores is a fact, as they all have great ability to identify patients with a high probability of deterioration (7, 10, 15–17).

There are no differences based on which to choose a score over another, as they have all been shown to have excellent predictive value for short-term complication, based on they all show a good adequacy. Nonetheless, we can propose NEWS/NEWS2 as the most suitable for general EMS use, because it is a simple and useful tool, validated for outpatient application, and indicated by scientific evidence for all levels of healthcare (7, 12, 13, 27). There are also two analytical parameters, easily determinable with portable analysers, which can support NEWS prediction; these are the readings of capillary blood glucose (21) and lactic acid in venous blood (12), which are easy to obtain and can be applied to outpatient care. In any case, in addition to determining the warning score, it is essential to carry out a clinical assessment of the patient (15), as these two procedures are complementary and objective and to be used together to determine clinical deterioration and the best response to the actual situation of the patient (13).

Even though all the scores we reviewed measure basically the same physiological parameters and have a very similar prediction capacity, the NEWS score is the most applied, which could be the reason why it was one of the first to be implemented at a national level, in this case the United Kingdom, in all health care areas. It provides some clinical advantages, such as assessing oxygen administration to the patient, and it is endorsed by the Royal College of Physicians (13). Specifically, the parameters that need to be recorded for the calculation of the NEWS 2 scale are respiratory rate, oxygen saturation, oxygen supply to the patient, heart rate, systolic blood pressure, temperature and neurological status by means of a simple assessment: AVDN.

The effectiveness of early detection scales is a fact, all of them having a great capacity to identify patients with a high probability of deterioration. In addition, they also appear to be effective in detecting patients who are not at risk of deterioration.

### Limitations

The main limitation is that there are a multitude of references for the use of early warning scores to predict serious risk in hospital settings, since they were specifically designed for this purpose. There is increasingly more research about their application in emergency and outpatient medicine, but it is still scarce. Finding bibliography on the validation of different scores for outpatient use turned out to be very complicated.

## Conclusions

The NEWS2 score is the most widespread and recognized in the world. This is because it is simple and easy to use by the whole clinical staff, including validation, effectiveness, and availability anywhere, and useful in triage and systematic patient assessment. Although we do recommend it, all the scores analyzed show great effectiveness for short-term (24 or 48 h) mortality prediction. The application of EWS in outpatient medicine can help standardize patient assessment and detect early clinical deterioration, this being one of the main EMS objectives, as it will lead to better quality patient care with lower morbidity and mortality. The scores suitable for prehospital use should be easy to calculate and not require large diagnostic means, as the latter are not available in outpatient care. Nonetheless, these scores can never replace clinical patient assessment, as the two must complement each other. NEWS/NEWS2 is the most effective validated early warning score in outpatient settings as far as the risk of complications and the detection of potentially serious emergency care situations. It is one of the first scores to have been implemented, easy to calculate and manage, and validated in both in-hospital and outpatient settings.

## Data Availability Statement

The raw data supporting the conclusions of this article will be made available by the authors, without undue reservation.

## Author Contributions

AB-E, VG-C, and RJ-V: conception or design of the work and final approval of the version to be published. AB-E and IS-A: data collection. VG-C, MC-H, and EM-S: data analysis, interpretation, and drafting the article. PM-M, AS-G, MG-L, JS-G, and JG-C: critical revision of the article. All authors contributed to the article and approved the submitted version.

## Conflict of Interest

The authors declare that the research was conducted in the absence of any commercial or financial relationships that could be construed as a potential conflict of interest.

## Publisher's Note

All claims expressed in this article are solely those of the authors and do not necessarily represent those of their affiliated organizations, or those of the publisher, the editors and the reviewers. Any product that may be evaluated in this article, or claim that may be made by its manufacturer, is not guaranteed or endorsed by the publisher.
