# Supplementary material for: Effectiveness of Early Warning Scores for Early Severity Assessment in Outpatient Emergency Care: A Systematic Review
Source: Front Public Health. 2022 Jul 14;10:894906. doi: 10.3389/fpubh.2022.894906 (PMC9330632; doi:10.3389/fpubh.2022.894906)
Supplement: Supplementary file 1 [file Table_1.pdf]

ANNEX 1: Parameters analyzed in the evaluated scales.

	BF	O2 SAT	Fi O2	HR	SBP	T	AVPU	DIURESIS	AGE	TRAUMA	GLUCOSE	LACTATE	GCS
EWS	√	√		√	√	√	√						
NEWS	√	√		√	√	√	√						
NEWS 2	√	√	√	√	√	√	√						
NEWS2-gluc	√	√	√	√	√	√	√				√		
NEWS2-L	√	√	√	√	√	√	√					√	
MEWS	√			√	√	√	√	√					
PMEWS	√	√		√	√	√	√						
MEWS GCS	√			√	√	√							√
ViEWS	√	√	√	√	√	√	√						
PhViEWS	√	√	√	√	√	√	√						
AbViEWS	√	√	√	√	√	√							
REMS	√	√		√	√		√		√				
MREMS	√	√		√	√				√				√
SWES	√	√		√	√	√	√						
TREWS	√			√	√	√	√			√			
HEWS	√	√	√	√	√	√	√						
PRS	√	√		√	√		√						
nzPHEWS	√	√	√	√	√	√	√						
WPSS	√	√		√	√	√	√						
PI	√			√	√		√						
RAPS	√			√	√								√
P. GOODACRE	√	√		√	√				√				√
P. GROARKE	√	√		√	√	√	√						
GAP					√				√				√
VSS	√	√		√	√								√
VSG	√	√		√	√	√							√

EWS: Early Warning Score

NEWS 2 (NEWS): National Early Warning Score

NEWS-gluc: National Early Warning Score + glucose

NEWS2-L: National Early Warning Score + lactate

MEWS: Modified Early Warning Score

PMEWS: Pandemic Medical Early Warning Score

MEWS GCS: Modified EWS with Glascow Coma Scale

ViEWS: VitalPac Early Warning Score

PhViEWS: Prehospital applied VitalPAC™ Early Warning Score

AbViEWS: Abbreviated VitalPac Early Warning Score

REMS: Rapid Emergency Medicine Score
MREMS: Modified Rapid Emergency Medicine Score
SWES: Scottish Early Warning Score
TREWS: Triage Early Warning Score
HEWS: Hamilton Early Warning Score
PRS: Prehospital risk score
nzNEWS: New Zealand Prehospital Early Warning Score
WPPS: Worthing Physiological Scoring System
PI: Prehospital Index
RAPS: Rapid Acute Physiology Score
P. GOODACRE: Goodacre Score
P. GROARKE: Groarke Score
GAP: Glasgow Coma Scale-Age-Systolic Blood Pressure Score
VSS: Vital Sign Score
VSG: Vital Sign Group Scores
BF = Breathing Rate
O2 SAT = Oxygen Saturation
HR = Heart Rate
SBP = Systolic blood pressure
T = Temperature
GCS = Glasgow Coma Scale
